# Kurt Semm: A laparoscopic crusader

**DOI:** 10.4103/0972-9941.30686

**Published:** 2007

**Authors:** K. Bhattacharya

**Affiliations:** Department of Surgery, Subham Hospital and Diagnostic Centre, Cooch Behar, West Bengal, India

**Keywords:** Laparoscopic appendectomy, pelviscopy, semm hysterectomy

## Abstract

A brief biography of Kurt Semm, the pioneer of laparoscopic appendectomy and inventor of various laparoscopic instruments are done, with special reference to his struggle to establish the foundation of minimally invasive surgery amongst his contemporary surgeons.

Kurt Karl Stephan Semm was born on March 23^rd^, 1927 in Munich, Germany to Margarate and Karl Semm. He received his medical degree from the Ludwig-Maximilians University School of Medicine in 1951. A specialist of Obstetrics and Gynaecology, he joined the International Fertility Association in 1953. In the early 1960s, Kurt Semm, as a talented University Assistant, started to dedicate his life to laparoscopy. He preferred the term ‘pelviscopy’ for operative laparoscopy.[1] He used it to differentiate between gynecological laparoscopy and procedures that other specialties performed for upper abdominal screening and liver biopsy. Based on his training both as an instrument maker and physician, his first inventions were to develop an electronic CO_2_ insufflator, a uterine manipulator and a tubal patency testing device.[2] He presented his work at the German, Austrian, and Swiss gynecological meeting in the early 1960s. Raoul Palmer, an American surgeon, work in France stimulated Semm's interest in gynecological laparoscopy, leading to his invention of CO_2_ pneu-automatic insufflator. In 1956, he founded the German Society of Fertility and Sterility. In the 1970s, Semm developed thermocoagulation, adapted the Roeder loop, and further invented extra- and intra-corporeal endoscopic knotting to achieve endoscopic hemostasis.

Semm's technique, however, was often criticized and insulted in public places, by his colleagues. Once, when he was making a slide presentation on ovarian cysts, ‘suddenly the projector was unplugged, with the explanation that such unethical surgery should not be presented’.[3] In 1970, after Semm became the Chairman of Obstetrics and Gynaecology at the University of Kiel, his co-workers demanded that he should undergo a brain scan because ‘only a person with brain damage would perform laparoscopic surgery’. In 1972, following Semm's presentation on laparoscopic ovarian cyst enucleation, a German gynecological professor remarked - ‘My young colleague, if you wish to advance in the German academic world, do not pay any attention to Semm's non-sense.’ Between 1975 and 1980, his idea to perform laparoscopic cholecystectomy was rejected by the general surgeons. The surgeons told him that they had enough work to do repairing the intestinal damage, which occurred during extensive laparoscopic adhesinolysis.

In 1981, Semm performed the first laparoscopic appendectomy. Following his lecture on laparoscopic appendectomy, the President of the German Surgical Society wrote to the Board of Directors of the German Gynecological society suggesting suspension of Semm from medical practice. Subsequently, Semm submitted a paper on laparoscopic appendectomy to the American Journal of Obstetrics and Gynecology, which was rejected as unacceptable for publication on the ground that the technique reported on was ‘unethical.’

The first publication on diagnostic laparoscopy by Raoul Palmer appeared in the early 1950s, followed by the publication of Frangenheim and Semm. Hans Lindermann and Kurt Semm practised CO_2_ hysteroscopy during the mid-seventies. In 1981, Professor Jon Beerman, President of the American Society of Reproductive Medicine, from Detroit, Michigan, visited Kurt Semm to see the ‘magic surgery.’ In the operation theatre, Beerman observed laparoscopic adnexectomy. He later commented ‘All I wanted to see was the reality of this surgery. Now, I am ready to go on my planned hunting trip.’ These comment in the US helped to make laparoscopic surgery acceptable in that part of the globe.

At a European meeting in Northern Italy, after a lecture of Kurt Semm on operative laparoscopy, Jordan Phillips, Director of the American Association of Gynecologic Laparoscopists, accused Semm of taking the technique to the absurd and of not even being recognized in his own country. Later, however, Phillips became a close friend of Semm and withdrew his words. From 1986 onward, Jordan Phillips organized 76 laparoscopic surgical training courses for Semm and his team throughout the US.

Semm established several standard procedures that were regularly performed, such as ovarian cyst enucleation, myomectomy, treatment of ectopic pregnancy and finally laparoscopic-assisted vaginal hysterectomy (nowadays termed as Cervical intra-fascial Semm hysterectomy). He also developed a medical instrument company Wisap in Munich, Germany, which still produces various endoscopic instruments of high quality. In 1985, he constructed the pelvi-trainer = laparo-trainer, a practical surgical model whereby colleagues could practice laparoscopic techniques. Semm published over 1000 papers in various journals.[4] He also produced over 30 endoscopic films and more than 20,000 colored slides to teach and inform interested colleague about his technique. His first atlas on pelviscopy and hysteroscopy was published in 1976, a slide atlas on pelviscopy, hysteroscopy, and fetoscopy in 1979, and his books on gynecological endoscopic surgery in German, English and many other languages in 1984, 1987, and 2002.

Finally in 2002, Semm received the ‘Pioneer in endoscopy’ award from the Board of Governors of the Society of American Gastrointestinal Endoscopic Surgeons (SAGES). In 2003, he received the honorary membership of the International Society of Gynecological Endoscopy in Mexico.

Semm loved sailing and flying. At the age of 76, he died due to Parkinson's disease in 2003, leaving behind his wife, Iseult O’ Neile and two children.
